# Still Stratus Not Altocumulus: Further Evidence against the Date/Party Hub Distinction

**DOI:** 10.1371/journal.pbio.0050154

**Published:** 2007-06-12

**Authors:** Nizar N Batada, Teresa Reguly, Ashton Breitkreutz, Lorrie Boucher, Bobby-Joe Breitkreutz, Laurence D Hurst, Mike Tyers

## Abstract

Analysis of multi-validated protein interaction data reveals networks with greater interconnectivity than the more segregated structures seen in previously available data. To help visualize this, the authors draw comparisons between continuous stratus clouds and altocumulus clouds.

Using a small dataset of protein–protein interactions [1], it was proposed that the yeast protein interaction network is made up of two sorts of hubs, party and date, and that these define modularity in the yeast protein interaction network. We found [2], by using several larger high-confidence datasets and appropriate statistical analyses, that we could not support these conclusions. Bertin et al. now invite analysis of a further dataset of protein-protein interactions, which they argue support the party/date distinction. The claimed properties of party and date hubs are not, however, present in this dataset either. In particular, when controlling for important covariables where necessary, there is no evidence for (1) bimodality in partner co-expression, (2) enrichment for similarly localized proteins that physically interact with party hubs, (3) a lower rate of evolution of party hubs, (4) differences in the effects of deletion of date and party hubs, or (5) higher genetic connectivity of date hubs. In sum, all of our prior conclusions remain robust and there is no evidence for distinctive classes of network hubs.

It was suggested [1] that some hub proteins operate at the same intracellular place and time with their multiple interactants (as if at a party) while others operate on a one-by-one basis with their numerous partners (as if on a date). Is this distinction informative? Originally, four features were used to the draw a partition between date and party hubs: expression bimodality, localization entropy, network fragmentation, and genetic connectivity [1]. A subsequent analysis suggested a fifth distinction, namely different rates of evolution after control for covariables [3]. Given the small size of the original dataset and the absence of statistical support for some of the assertions, we asked [2] whether these claims were robust. In both the original dataset and in new high-confidence interaction datasets [2], we found we could not support any of the five points of evidence.

Bertin et al. now nominate a new dataset, which they argue supports three of the five points of evidence. Bertin et al. first note a curation issue with one of our many datasets, called HC, which inadvertently contains interactions that were, owing to an ambiguity in the literature, supported by a single analysis. We certainly agree that inclusion of the data from [4] and [5] as independent validations was in error, as the data in [5] indeed fully encompasses that of [4] (A-C Gavin, personal communication). However, approximately half of the interactions reported in [4] remain multi-validated by other means. An updated high-confidence dataset that removes this duplication and incorporates more recent interaction data is available here (Dataset S1) and as a download from the BioGRID database (see http://www.thebiogrid.org). Importantly, however, our dataset HC^m^ is unaffected by the above concern as we required validation of an interaction by multiple different methods. The new build of Bertin et al. (called “filtered-HC”) mimics HC^m^ by excluding interactions not multivalidated with different methods. As the results of HC^m^ confirmed those of our other datasets [2], we were surprised by the claim that the date/party distinction is still supported in filtered-HC. Because this dataset provides the most robustly defendable set of interactions, here we re-analyse the filtered-HC network to ask whether it substantiates the date/party distinction.

## No Evidence for Bimodality of Co-Expression Values

Han et al. originally proposed that clear evidence for a binary hub classification (party versus date) derives from bimodal distribution of co-expression (PCC) values: one class with high average PCCs (party hubs) and the other with low average PCCs (date hubs) [1]. This proposal was based exclusively on visual inspection of the data. By contrast, we applied a formal test that examines deviation from a null of unimodality [6,7] and found no evidence for bimodality. Up to now we have analysed 25 expression datasets across seven protein interaction builds (including filtered-HC; Table 1) and added datasets nominated by Han et al., a total of 181 separate tests. To a first approximation, by chance we should expect to see around nine incidences of significance at the 5% significance level owing to type I error (although this assumes independence between datasets). We find just two.

**Table 1:**
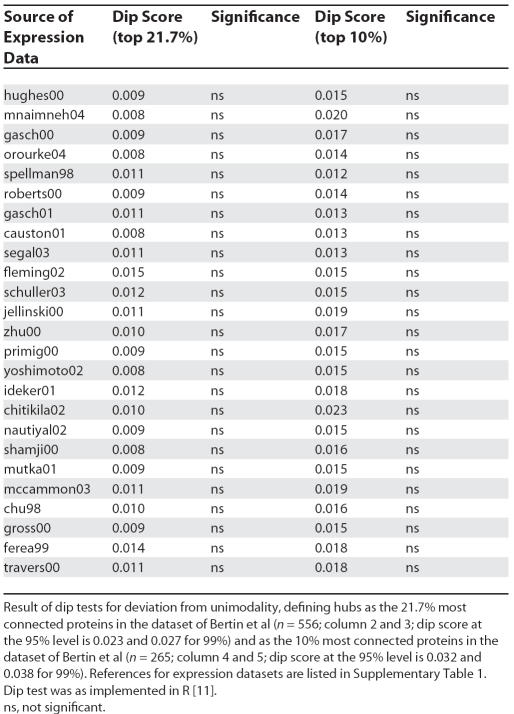
Test for Bimodality of Neighbour Correlation Distribution at Two Different Hub Connectivity Thresholds

Given this lack of evidence for bimodality, Bertin et al. appear to concur that bimodality cannot be used to define party and date hubs. Surprisingly though, they now assert that bimodality never was a key point of evidence. The original definition of date and party hubs, however, stated that bimodality represented a “natural boundary” between the two classes [1]; indeed it was argued that the lack of obvious bimodality in some expression datasets was due to low sample sizes [1]. At the same time, Bertin et al. also venture to suggest that the standard statistical test for deviation from unimodality [6,7] has a high false negative rate. It does not (see Text S1).

## Neighbours of Date Hubs Do Not Have more Diverse Localizations

Originally, Han et al. reported that the partners of date hubs have more diverse intracellular localizations, as measured by information entropy [1]. However, this analysis did not normalise for connection density and arbitrarily omitted data from some cellular compartments [1]. In the filtered-HC dataset again (Table 2), as before [2], upon normalization and inclusion of all the data, the entropy is in the opposite direction to that predicted by the date/party hypothesis. This inversion we showed [2] is owing to differences in abundance that follow from the assignment of party hubs as those with highly co-expressed partners (PCC > 0.5). As Bertin et al. make no statement on this issue, we assume that they do not dispute this result.

**Table 2:**
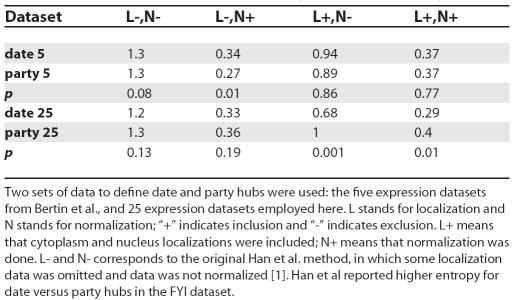
Opposite Localization Entropy of Date and Party Hubs

## Definitions and Inferences

The evidence for the biological relevance of date and party hubs falls into two classes: the definitional, namely bimodality/co-expression and subcellular colocalization, and the inferential, or corollary behaviours that may derive from the underlying biology. As the definitional aspects do not bear scrutiny, one must be suspicious that any correlates are merely consequences of the method used to define the two hub classes. The only standing criterion left is the arbitrary distinction between those hubs with a PCC > 0.5 and those without. Highly co-expressed proteins do have a number of odd properties, namely higher connectivity and abundance. These biases are robust in the filtered HC dataset: party hubs have higher connectivity (*p* = 0.00006) and protein abundance (*p* = 0.001). It is then important to ask whether further properties stem from such biases.

## Given their Abundance, Date and Party Hubs Do Not Evolve at Different Rates

Bertin et al. find that party hubs evolve more slowly. As originally noted [3], the question is whether party hubs evolve slower than date hubs when controlling for important covariates, most notably protein abundance [8]. We showed previously that any weak tendency for party hubs to evolve slower was accounted for by their abundance [2]. Unlike our prior analysis, Bertin et al. do not ask if party and date hubs evolve at different rates controlling for abundance but, instead, ask if PCC is related to evolutionary rate controlling for abundance. However, they inappropriately apply a parametric test (Pearson product-moment correlation) that requires the distribution of all variables to be normally distributed. Although the method is robust to some degree of deviation from normality, the extent to which the abundance data is non-normal is extreme (Shaprio-Wilks tests for null of normality, *W* = 0.2, *p* << 0.0001, *W* = 1 implying normality, *W* << 1 implying deviation from normality). This leaves two avenues: either to transform the data to make them approximately normal or to perform the equivalent non-parametric test.

Partial Spearman's correlation is the nonparametric equivalent. Using evolutionary rate data from *sensu strictu* yeasts [9], controlling for abundance [10], the more highly co-expressed genes have, if anything, a slightly higher rate of evolution (partial rho controlling for abundance, *rho* = +0.13, *p* = 0.02, *p* determined by simulation, implemented in R [11]). If we log transform the abundance data then the parametric correlation agrees that the sign of the partial correlation changes (*rho* = +0.029, *p* = 0.36). The log transformed abundance data has a Shapiro Wilks *W* score of 0.95, as opposed to 0.2 for the untransformed.

**Figure 1:**
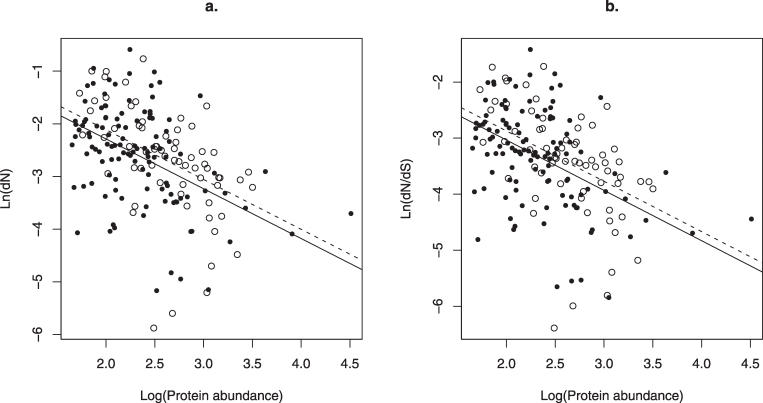
No Difference in the Evolutionary Rate of Date Hubs and Party Hubs ANCOVA of natural log of rate of evolution, measured either as (A) *dN* or (B) *dN/dS* predicted by date/party distinction, with protein abundance as the covariate. The black line is for date, the dotted for party hubs. For (A), ANCOVA ln(*K*a) versus party/date with log10(abundance) as a covariate: effect of covariate, *t* = 6.9, *p* ~ 8 x 10–11, effect of date/party, *t* = 1.27, *p* = 0.21. For (B), ANCOVA ln(*K*a/*K*s) versus party/date with log10(abundance) as a covariate: effect of covariate, *t* = 6.99, *p* ~ 5 x 10-11, effect of date/party, *t*=1.24, *p* = 0.21. Note that taking the log of the variables on the y-axis forces loss of two data points (one party, one date) with *dN* = 0. However, results are unaffected by using, for example, ln(0.1 + *dN*) and ln(0.1 + *dN/dS*), which permits their inclusion. Similarly the residuals from the fit of *x* versus *y* are no different for date and party hubs for ln(0.1 + *dN*) residuals of date are if anything lower than those for party (-0.026 versus 0.46 but not significantly so, *p* = 0.16, *t*-test; ln(0.1 + *dN/dS*) mean for date is −0.015, for party 0.027, *p* = 0.18). We have repeated the analysis using a different outgroup (S. bayanus), and still find no effect on covariate controlled analysis (unpublished data).

Our previous tests differed from that performed by Bertin et al: we employed analysis of covariance (ANCOVA) to ask whether date and party hubs evolve at different rates when covariate controlled (this being the prior claim [3]). We find, in accord with our results [2], that differences in abundance explain all difference in rates of evolution between the two classes (Fig 1). In the ANCOVA, as above, if anything date hubs evolve slightly slower than party hubs (Fig 1). Analysis of residuals supports these results (Fig 1). Although we can recover the result of Bertin et al. when Pearson's partial correlation is inappropriately applied to nontransformed data, all appropriate tests reject the contention of evolutionary rate differences.

Bertin et al. also suggest that a recent study of hub proteins that bind partners at multiple different sites, as opposed to re-use of the same site, provides support for the difference in evolutionary rate between party and date proteins [12]. However, this report failed to properly control for abundance [12], which if performed reveals no differences (*p* > 0.45) (Text S2). These results accord with our prior finding that, controlling for abundance, more highly connected hubs do not evolve more slowly, in no small part owing to re-use of binding sites [13]. In summary, evolutionary rate differences provide do not support the date/party distinction.

## No Evidence for Large Differences in Effects of Hub Deletion when Allowing for Connectivity

It is argued that date hubs establish network integrity because of their positioning as intermodule linkers, as opposed to the intramodule positioning of party hubs [1]. But might any differences in deletion of date versus party hubs merely reflect a difference in connectivity of the two hub classes? Two metrics were used to measure the effect of hub deletion on the network: characteristic pathway length (CPL) and main component size (MCS) [1]. We previously considered [2] CPL to be of limited worth, because differences in pathway length may not have biological consequences (for example, since diffusion is fast, transmission delays due to increase in number of intermediate steps may be inconsequential). Moreover, CPL is susceptible to network incompleteness, which is acute for small stringent datasets such as filtered-HC. However, to enable comparison with Bertin et al., we analyze both MCS and CPL.

**Figure 2:**
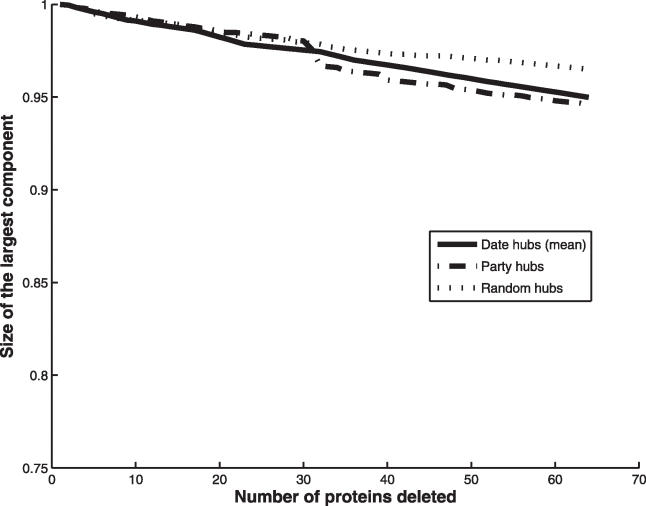
Effect of Hub Deletion Controlling for Connectivity via Hub Deletion for Nonessential Party and Date Hubs Date and party hub deletion effect on the integrity of the interaction network as measured by the relative size of the largest connected component (MCS) after deletion. Hubs were deleted in descending order by connectivity. Because the number of date hubs was much larger than number of party hubs (189 versus 64 respectively), we sampled the same number of date hubs as party hubs 200 times and determined the deletion effect each time. The mean effect of deletion of date hubs is plotted.

**Figure 3:**
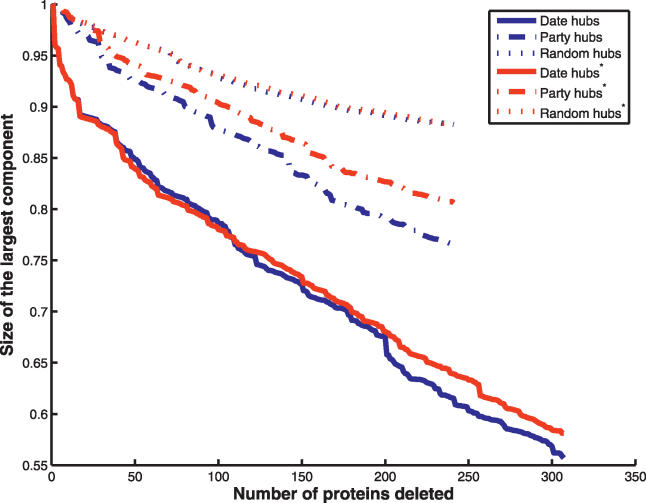
Effect of Hub Deletion Controlling for Connectivity via Hub Deletion before and after Random Swap of Date and Party Hubs of Similar Connectivity Lines in red are after 50% swap of hubs, in blue for the original case. Because hub swapping has no effect, connectivity (not position in the network) explains why date and party have apparently different effects upon deletion.

In addition to connectivity, it is desirable to correct for dispensability, because it is not biologically sensible to analyze networks that are fatally crippled by the loss of essential genes. Fortunately, nonessential date and party hubs have equal connectivity (*p* = 0.94), and thus control for both parameters simultaneously. Deletion of nonessential date and party hubs has an identical effect on network integrity (for MCS, see Figure 2; for CPL see Figure S1). Bertin et al. observe the same result for MCS even without controlling for dispensability. As an alternative means to correct for connectivity, we randomly swapped date and party hubs of the same connectivity. If the differential deletion effect is solely due to inter-versus intramodule positioning, then interchanging date with party hubs should obviate the difference. Instead, hub swapping yields the same deletion profile as the original unswapped case (Figure 3). Finally, we asked whether, even in the absence of controls for connectivity or dispensability, the difference between party and date hubs is sensitive to removal of just a few extreme hubs. Removal of just the top two percent of hubs obviates the difference between date and party hub deletion on MCS (Figure S2).

In sum, controlling for connectivity by two different means eliminates the difference between date and party hub deletion; even when not controlling for connectivity, the deletion effect relies entirely on a few extreme date hubs. There is thus no reason to suppose date and party hubs have different network positions.

## No Evidence for a Difference in Genetic Connectivity

**Figure 4:**
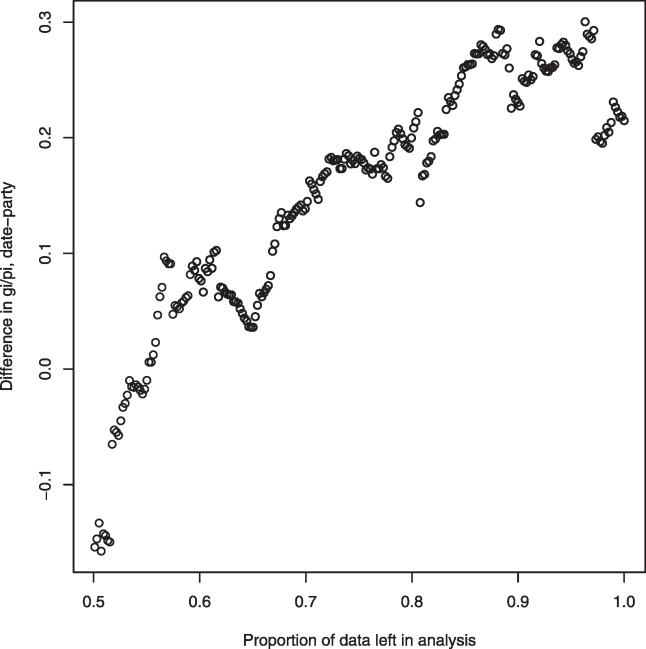
The Effect of Study Bias on the Difference between the Mean Number of Genetic Interactions per Physical Connection (*g*_i_ */p*_i_) of Party and Date Hubs We define bias as the difference between the number of independent validations of a genetic interaction of a given protein and the actual, nonredundant genetic connectivity, normalised by the nonredundant genetic connectivity [14]. We rank ordered all genes according to their study bias. We then eliminated the most biased data point and recalculated the *g* *_i_*/*p* *_i_* difference in for date versus party hubs for the remaining genes (reported on the *y*-axis). We then removed the next most biased, and so forth. At 0.5 residual, half of the original 489 genes were left in the analysis. Purging of the most biased genes removes any tendency for party and date hubs to differ; any possible difference between party and date hubs is hence owing to study bias.

While Bertin et al. contend that date hubs have more genetic interactions in filtered-HC, they acknowledge that study bias may confound analysis, as noted [1]. Using a metric of study bias [14] (see Figure 4), we find that date and party hubs do indeed differ in their study bias (*p* = 0.039, Mann Whitney *U*-test). To examine the impact of this, we considered the difference in mean number of genetic interactions per physical connection (*g*_i_/*p*_i_) between date and party hubs; this metric controls for the fact that genetic and physical interactions are positively correlated [15]. As we incrementally purge the data of study bias, the difference in mean *g*_i_/*p*_i_ between date and party hubs diminishes to zero (Figure 4). Even making no allowance for study bias, *g*_i_/*p*_i_ for date and party hubs is not significantly different (Mann Whitney *U*-test; *p* > 0.06). There is thus no significant difference in genetic connectivity of party and date hubs.

## Conservation of Date/Party Classification Is a Consequence of Definition, Not of Biology

Finally, Bertin et al. raise one new prospective line of evidence, namely, those proteins that appear as hubs across datasets tend to preserve their status as party or date. This observation, however, follows definition: if a hub is co-expressed (with PCC > 0.5) in any one dataset, it is defined as a party hub; if not, by default it is a date hub. Once a hub is classified as a party hub, its status cannot change solely with the addition of extra expression data. The reverse classification, i.e., date to party, is also disfavored because co-expression across different assays is not independent. The low rates of transfer of hub status merely follow from definitions and do not address the biological validity of the date/party distinction.

## Summary

In the new filtered-HC dataset, as in others, the two definitional criteria of date/party hubs find no support. Four corollary points of evidence— rate of evolution, effect of deletion on network topology, genetic connectivity, and hub status quo—also find no support. That across multiple datasets, and under multiple different tests, we repeatedly find no evidence for the date/party hypothesis suggests that network hubs do not fall into discrete classes.

## Supporting Information

Figure S1 Deletion of Nonessential Date and Party Hubs Does Not Have a Differential Effect on CPL in the Filtered-HC Dataset Each indicated hub class was serially deleted in order of decreasing connectivity (percent of network deleted, *x*-axis) and CPL calculated after each deletion (*y*-axis) Deletion of either all hubs, date hubs, party hubs, or random nodes recapitulates previously reported effects on CPL. However, as for MCS analysis described in the main text, when connectivity is controlled for by deletion of only nonessential hubs, there is no differential contribution of date versus party hubs to CPL. (1.1 MB EPS).

Figure S2 Effect of Hub Deletion before and after Removal of the Top 2% most Highly Connected Proteins in the Filtered-HC Network Lines in blue are those prior to removal of top 2%, in red after removal. Note that the difference between date and party is very sensitive to the presence of very few extremely highly connected proteins, most of which are classified as date hubs.
(16 KB EPS).

Table S1 References for the Expression Data Used (28 KB DOC).

Text S1 Testing the False Negative Rate of the Dip Test (20 KB DOC).

Text S2 No Difference in the Rate of Evolution of Singlish and Multi-Proteins when Controlling for Abundance (25 KB DOC).

Dataset S1 Updated High-Confidence Interaction Dataset (2 MB TXT)

## References

[pbio-0050154-b001] Han JD, Bertin N, Hao T, Goldberg DS, Berriz GF (2004). Evidence for dynamically organized modularity in the yeast protein-protein interaction network. Nature.

[pbio-0050154-b002] Batada NN, Reguly T, Breitkreutz A, Boucher L, Breitkreutz BJ (2006). uStratus not altocumulus: A new view of the yeast protein interaction network. PLoS Biol.

[pbio-0050154-b003] Fraser HB (2005). Modularity and evolutionary constraint on proteins. Nat Genet.

[pbio-0050154-b004] Gavin AC, Bosche M, Krause R, Grandi P, Marzioch M (2002). Functional organization of the yeast proteome by systematic analysis of protein complexes. Nature.

[pbio-0050154-b005] Gavin AC, Aloy P, Grandi P, Krause R, Boesche M (2006). Proteome survey reveals modularity of the yeast cell machinery. Nature.

[pbio-0050154-b006] Hartigan JA, Hartigan PM (1985). The dip test of unimodality. Ann Stat.

[pbio-0050154-b007] Hartigan PM (1985). Computation of the dip statistic to test for unimodality. J Roy Stat Soc C, App Stat.

[pbio-0050154-b008] Drummond DA, Bloom JD, Adami C, Wilke CO, Arnold FH (2005). Why highly expressed proteins evolve slowly. Proc Natl Acad Sci U S A.

[pbio-0050154-b009] Hirsh AE, Fraser HB, Wall DP (2005). Adjusting for selection on synonymous sites in estimates of evolutionary distance. Mol Biol Evol.

[pbio-0050154-b010] Newman JR, Ghaemmaghami S, Ihmels J, Breslow DK, Noble M (2006). Single-cell proteomic analysis of S. cerevisiae reveals the architecture of biological noise. Nature.

[pbio-0050154-b011] R Development Core Team (2005). R: A language and environment for statistical computing.

[pbio-0050154-b012] Kim PM, Lu LJ, Xia Y, Gerstein MB (2006). Relating three-dimensional structures to protein networks provides evolutionary insights. Science.

[pbio-0050154-b013] Batada NN, Hurst LD, Tyers M (2006). Evolutionary and physiological importance of hub proteins. PLoS Comp Bio.

[pbio-0050154-b014] Reguly T, Breitkreutz A, Boucher L, Breitkreutz B-J, Hon G (2006). Comprehensive curation and analysis of global interaction networks in Saccharomyces cerevisiae. J Biol.

[pbio-0050154-b015] Ozier O, Amin N, Ideker T (2003). Global architecture of genetic interactions on the protein network. Nat Biotechnol.

