# Supplementary material for: Still Stratus Not Altocumulus: Further Evidence against the Date/Party Hub Distinction
Source: PLoS Biol. 2007 Jun 12;5(6):e154. doi: 10.1371/journal.pbio.0050154 (PMC1892831; doi:10.1371/journal.pbio.0050154)
Supplement: Supplementary file 1 [file pbio.0050154.sd001.doc]

# Supplementary Information 1. Testing the false negative rate of the dip test.

Could it be that the dip-test is not powerful enough to detect instances of bimodality? That is, does the dip test have a high false negative rate? To address this we consider simulations in which we mimic bimodal distributions. For the date hubs we assume a mean co-expression of zero. Han et al arbitrarily assert that date hubs cannot have co-expression correlation greater than 0.5 so we chose this as the 99% upper limit by setting the standard deviation of our simulant normal distribution to be 0.5/3. For the party hubs we consider a series of mean co-expression scores (0.6, 0.65, 0.7, 0.75). In each case the standard deviation is adjusted to ensure 99% bounds do not go below 0.5. As the dip test is expected to be least powerful when the standard deviation is higher, to be conservative we assume that the standard deviation is at it uppermost limit, i.e. s.d. = (mean-0.5)/3. Any randomly generated number greater than 1 or less than –1 is set to 1 and –1 respectively. For each simulant dataset we apply the dip test and ask whether it rejects the null of unimodality. For each set of parameter values we produce 1000 simulations. The definition employed by Han et al. permits a party hub to be considered as such if it appears in the upper mode of a distribution in just one test. Hence in any given data set the proportion of proteins that are in the putative upper mode may well be less than the total (over 30%). The method correctly reports significance 100%, 100%, 99.99% and 79% of the time if 30%, 20%, 15% and 10% respectively are party hubs. Thus the dip-test has a low false negative rate.
