# Supplementary material for: Still Stratus Not Altocumulus: Further Evidence against the Date/Party Hub Distinction
Source: PLoS Biol. 2007 Jun 12;5(6):e154. doi: 10.1371/journal.pbio.0050154 (PMC1892831; doi:10.1371/journal.pbio.0050154)
Supplement: Supplementary file 2 [file pbio.0050154.sd002.doc]

**Supplementary Information 2. No difference in the rate of evolution of singlish and multi-proteins when controlling for abundance**

It is claimed that that multibinding proteins have a lower rate of evolution than singlish ones [1], this being employed by Bertin et al. as support for the date-party distinction. Using the supplied classification of multi-binding and singlish proteins (supplementary table 6 of [1]) we can indeed repeat the finding that multibinding proteins have a lower Ka and lower Ka/Ks than singlish ones (Ka: P=0.017, Ka/Ks: 0.012; Mann Whitney U test). However the prior report [1] fails to properly control for protein abundance as a covariate. Multibinding proteins have, however, higher abundance (*P*=0.04, Mann Whitney U test) which could, in principle, explain their apparently lower rate of evolution, protein abundance being the strongest predictor of rates of protein evolution in yeast. We address this in two ways.

First, we have performed ANCOVA with Log(Abundance) being the covariate. We find that the claimed predictor of protein rates of evolution is explained by covariance with protein abundance (ANCOVA, Log(Ka+0.1) as a function of multi/singlish with Log (protein abundance) as a covariate, *P* for effect of multi-singlish = 0.53, *P* for effect of abundance=0.0009; ANCOVA, Log(Ka/Ks+0.1) as a function of multi/singlish with Log (protein abundance) as a covariate, *P* for effect of multi-singlish = 0.45, *P* for effect of abundance=0.0017).

Second, as an alternative to ANCOVA, we can consider the residuals of the plot of Log(Ka+0.1) against Log(Abundance). Again the singlish and multi proteins do not differ (P=0.81, Mann Whitney U test). Likewise for Log(Ka/Ks+0.1), *P*=0.87. These tests indicate that, controlling for abundance, there is no evidence that multi and singlish proteins differ in their rate of evolution, only in their abundance

1.Kim PM, Lu LJ, Xia Y, Gerstein MB (2006) Relating three-dimensional structures to protein networks provides evolutionary insights. Science 314: 1938-1941.
