# Supplementary material for: Still Stratus Not Altocumulus: Further Evidence against the Date/Party Hub Distinction
Source: PLoS Biol. 2007 Jun 12;5(6):e154. doi: 10.1371/journal.pbio.0050154 (PMC1892831; doi:10.1371/journal.pbio.0050154)
Supplement: Supplementary file 3 [file pbio.0050154.st001.doc]

**Supplementary Table 1. Expression data employed.**

| **Key** | **Reference** |
| --- | --- |
| hughes00 | Hughes, T. R., Marton, M. J., Jones, A. R., Roberts, C. J., Stoughton, R., Armour, C.  D., Bennett, H. A., Coffey, E., Dai, H., He, Y. D., Kidd, M. J., King, A. M., Meyer, M.   R., Slade, D., Lum, P. Y., Stepaniants, S. B., Shoemaker, D. D., Gachotte, D.,  Chakraburtty, K., Simon, J., Bard, M., and Friend, S. H.  Functional discovery via a compendium of expression profiles. Cell 2000 102, 109-26. |
| mnaimneh04 | Mnaimneh S, Davierwala AP, Haynes J, Moffat J, Peng WT, Zhang W, Yang X, Pootoolal J, Chua G, Lopez A, Trochesset M, Morse D, Krogan NJ, Hiley SL, Li Z, Morris Q, Grigull J, Mitsakakis N, Roberts CJ, Greenblatt JF, Boone C, Kaiser CA, Andrews BJ, Hughes TR.  Exploration of essential gene functions via titratable promoter alleles. Cell. 2004 Jul 9;118(1):31-44. |
| gasch00 | Gasch AP, Spellman PT, Kao CM, Carmel-Harel O, Eisen MB, Storz G, Botstein D, Brown PO.  Genomic expression programs in the response of yeast cells to environmental changes. Mol Biol Cell. 2000 Dec;11(12):4241-57. |
| orourke04 | O'Rourke SM, Herskowitz I.  Unique and redundant roles for HOG MAPK pathway components as revealed by whole-genome expression analysis. Mol Biol Cell. 2004 Feb;15(2):532-42. Epub 2003 Oct 31. |
| spellman98 | Spellman PT, Sherlock G, Zhang MQ, Iyer VR, Anders K, Eisen MB, Brown PO,  Botstein D, Futcher B.  Comprehensive identification of cell cycle-regulated genes of the yeast Saccharomyces cerevisiae by microarray hybridization. Mol Biol Cell. 1998 Dec;9(12):3273-97. |
| roberts00 | Roberts CJ, Nelson B, Marton MJ, Stoughton R, Meyer MR, Bennett HA, He YD,  Dai H, Walker WL, Hughes TR, Tyers M, Boone C, Friend SH.  Signaling and circuitry of multiple MAPK pathways revealed by a matrix of global gene expression profiles. Science. 2000 Feb 4;287(5454):873-80. |
| gasch01 | Gasch AP, Huang M, Metzner S, Botstein D, Elledge SJ, Brown PO (2001).  Genomic expression responses to dna-damaging agents and the regulatory role of the yeast ATR homolog Mec1p. Mol Biol Cell 2001 Oct;12(10):2987-3003. |
| causton01 | Causton, H. C., Ren, B., Koh, S. S., Harbison, C. T., Kanin, E., Jennings, E. G., Lee,  T. I., True, H. L., Lander, E. S., and Young, R. A.  Remodeling of Yeast Genome Expression in Response to Environmental Changes. Mol Biol Cell 2001 12, 323-337. |
| segal03 | Segal E, Shapira M, Regev A, Pe'er D, Botstein D, Koller D, Friedman N.  Module networks: identifying regulatory modules and their condition-specific regulators from gene expression data. Nat Genet. 2003 Jun;34(2):166-76. |
| fleming02 | Fleming JA, Lightcap ES, Sadis S, Thoroddsen V, Bulawa CE, Blackman RK.  Complementary whole-genome technologies reveal the cellular response to proteasome inhibition by PS-341. Proc Natl Acad Sci U S A. 2002 Feb 5;99(3):1461-6. |
| schuller03 | Schuller C, Mamnun YM, Mollapour M, Krapf G, Schuster M, Bauer BE, Piper PW, Kuchler K.  Global phenotypic analysis and transcriptional profiling defines the weak acid stress response regulon in Saccharomyces cerevisiae. Mol Biol Cell. 2004 Feb;15(2):706-20. |
| jellinski00 | Jelinsky, S. A., Estep, P., Church, G. M., and Samson, L. D.  Regulatory networks revealed by transcriptional profiling of damaged Saccharomyces cerevisiae  cells: Rpn4 links base excision repair with proteasomes. Mol Cell Biol 2000 20, 8157-67. |
| zhu00 | Zhu, G., Spellman, P. T., Volpe, T., Brown, P. O., Botstein, D., Davis, T. N., and  Futcher, B.  Two yeast forkhead genes regulate the cell cycle and pseudohyphal growth. Nature 2000 406, 90-4. |
| primig00 | Primig, M., Williams, R. M., Winzeler, E. A., Tevzadze, G. G., Conway, A. R., Hwang,  S. Y., Davis, R. W., and Esposito, R. E. .  The core meiotic transcriptome in budding yeasts. Nat Genet 2000 26, 415-23. |
| yoshimoto02 | Yoshimoto H, Saltsman K, Gasch AP, Li HX, Ogawa N, Botstein D, Brown PO, Cyert MS.  Genome-wide analysis of gene expression regulated by the calcineurin/Crz1p signaling pathway in Saccharomyces cerevisiae. J Biol Chem. 2002 Aug 23;277(34):31079-88. Epub 2002 Jun 10 |
| ideker01 | Ideker T, Thorsson V, Ranish JA, Christmas R, Buhler J, Eng JK, Bumgarner R, Goodlett DR.  Integrated genomic and proteomic analyses of a systematically perturbed metabolic network. Science 2001 May 4;292(5518):929-34 |
| chitikila02 | Chitikila C, Huisinga KL, Irvin JD, Basehoar AD, Pugh BF.  Interplay of TBP inhibitors in global transcriptional control. Mol Cell. 2002 Oct;10(4):871-82. |
| nautiyal02 | Nautiyal S, DeRisi JL, Blackburn EH.  The genome-wide expression response to telomerase deletion in Saccharomyces cerevisiae. Proc Natl Acad Sci U S A. 2002 Jul 9;99(14):9316-21. |
| shamji00 | Shamji, A. F., Kuruvilla, F. G., and Schreiber, S. L.  Partitioning the transcriptional program induced by rapamycin among the effectors of the Tor proteins. Curr Biol 2000, 10, 1574-1581. |
| mutka01 | Mutka SC, Walter P.  Multifaceted physiological response allows yeast to adapt to the loss of the signal recognition particle-dependent protein-targeting pathway. Mol Biol Cell. 2001 Mar;12(3):577-88. |
| mccammon03 | McCammon MT, Epstein CB, Przybyla-Zawislak B, McAlister-Henn L, Butow RA.  Global transcription analysis of Krebs tricarboxylic acid cycle mutants reveals an alternating pattern of gene expression and effects on hypoxic and oxidative genes. Mol Biol Cell. 2003 Mar;14(3):958-72. |
| chu98 | Chu S, DeRisi J, Eisen M, Mulholland J, Botstein D, Brown PO, Herskowitz I.  The transcriptional program of sporulation in budding yeast. Science. 1998 Oct 23;282(5389):699-705. Erratum in: Science 1998 Nov 20;282(5393):1421. |
| gross00 | Gross C, Kelleher M, Iyer VR, Brown PO, Winge DR.  Identification of the copper regulon in Saccharomyces cerevisiae by DNA microarrays. J Biol Chem. 2000 Oct 13;275(41):32310-6. |
| ferea99 | Ferea TL, Botstein D, Brown PO, Rosenzweig RF.  Systematic changes in gene expression patterns following adaptive evolution in yeast. Proc Natl Acad Sci U S A. 1999 Aug 17;96(17):9721-6. |
| travers00 | Travers KJ, Patil CK, Wodicka L, Lockhart DJ, Weissman JS, Walter P.  Functional and genomic analyses reveal an essential coordination between the unfolded protein response and ER-associated degradation. Cell. 2000 Apr 28;101(3):249-58. |
